# Bilateral Geyser Sign Secondary to Calcium Pyrophosphate Deposition Disease: A Rare Presentation

**DOI:** 10.7759/cureus.113957

**Published:** 2026-08-04

**Authors:** Susana Almeida, Anita Cunha, José Tavares-Costa, Filipa Teixeira, Francisca Guimarães

**Affiliations:** 1 Rheumatology, Unidade Local de Saúde do Alto Minho, EPE, Ponte de Lima, PRT

**Keywords:** acromioclavicular joint, calcium pyrophosphate deposition disease, geyser sign, rotator cuff tear, shoulder

## Abstract

The Geyser Sign (GS) is a rare imaging sign characterized by synovial fluid leakage through a full-thickness rotator cuff tear into the subacromial bursa and acromioclavicular joint (ACJ), resulting in capsular distension and cyst formation. We report the case of a 72-year-old man who presented with bilateral shoulder pain and a firm and compressible swelling over both ACJs with marked inflammatory signs. Radiographs demonstrated ACJ soft-tissue swelling, degenerative changes of the glenohumeral joint (GHJ) and ACJ, and features of chronic rotator cuff arthropathy. Computed tomography revealed severe omarthrosis, complete rotator cuff tear, glenohumeral effusion, subacromial bursal distension, and large ACJ cysts measuring 5.4 cm on the right and 6.3 cm on the left, confirming bilateral GS. Ultrasound-guided right ACJ cyst aspiration revealed viscous yellow synovial fluid with 14,822 white blood cells/mm³ (89% polymorphonuclear cells) and calcium pyrophosphate crystals, consistent with calcium pyrophosphate deposition disease. Triamcinolone injection resulted in symptomatic relief over six months of follow-up. Bilateral presentation of GS is uncommon, and calcium pyrophosphate crystals in these cysts are rarely reported despite their common occurrence in degenerative joints. Management strategies range from conservative to surgical, depending on symptoms, functional status, extension of rotator cuff tears, and comorbidities.

## Introduction

The Geyser Sign (GS) is a rare imaging sign characterized by a geyser-like appearance, typically resulting from a full-thickness rotator cuff tear and/or degenerative changes of the acromioclavicular joint (ACJ). First described by Craig in 1984 during fluoroscopy-guided shoulder arthrography [1,2], GS represents the passage of synovial fluid through a rotator cuff tear into the subacromial bursa, with subsequent extrusion through the ACJ, resulting in capsular distension and cyst formation. Two types of ACJ cysts have been identified: type 1, without communication with the glenohumeral joint (GHJ); and type 2, associated with a rotator cuff tear that allows fluid communication between the ACJ and GHJ [3,4]. We present a case of bilateral GS associated with calcium pyrophosphate deposition disease (CPPD), a presentation rarely described, as most reported cases of GS are unilateral and typically associated with advanced degenerative shoulder disease, and the identification of calcium pyrophosphate crystals within ACJ cysts remains exceptionally rare [4].

## Case presentation

**Figure 1:**
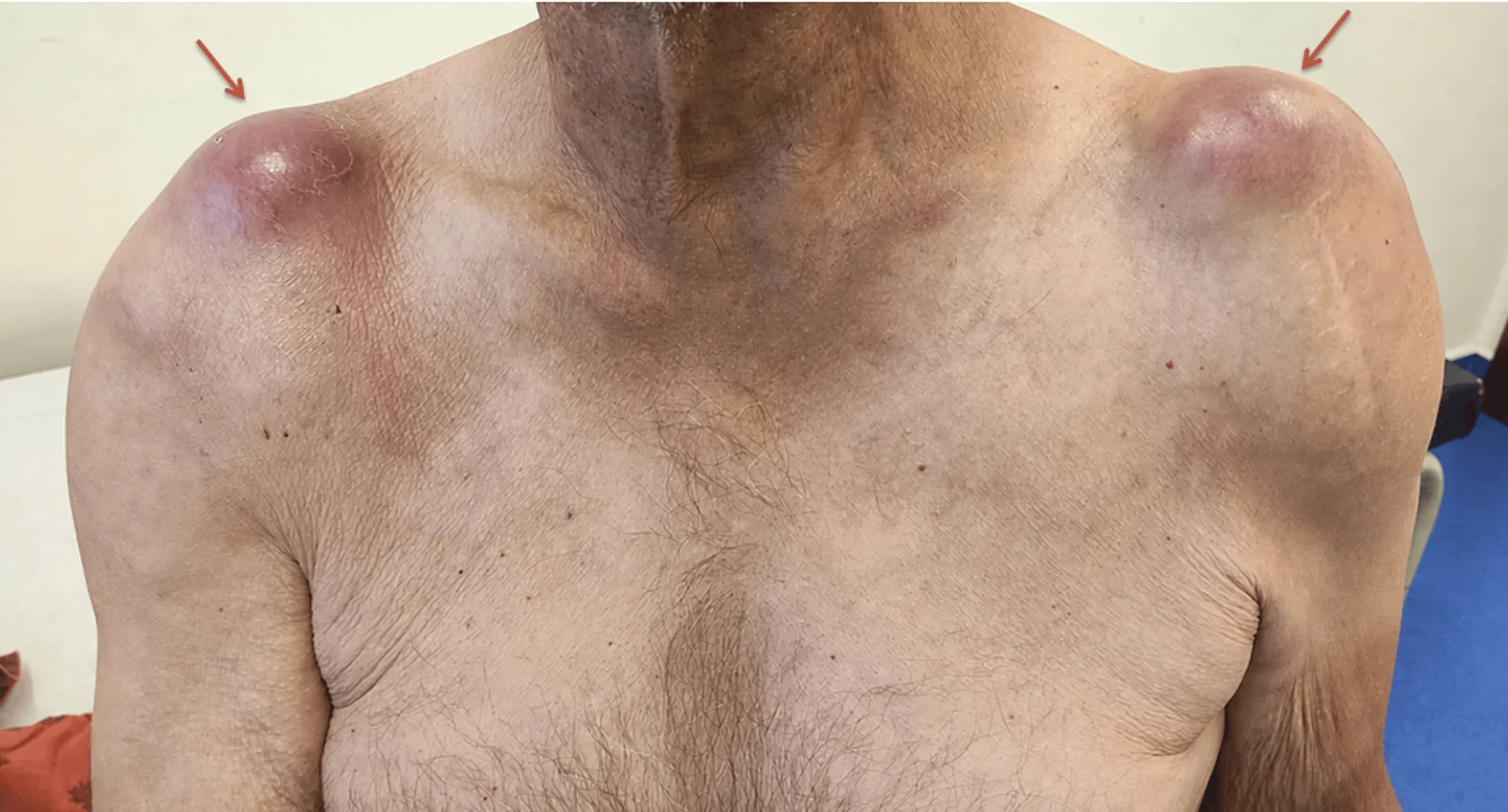
Clinical image of the shoulders showing bilateral prominent cystic swellings over the ACJs with inflammatory signs (arrows). ACJ, acromioclavicular joint

**Figure 2:**
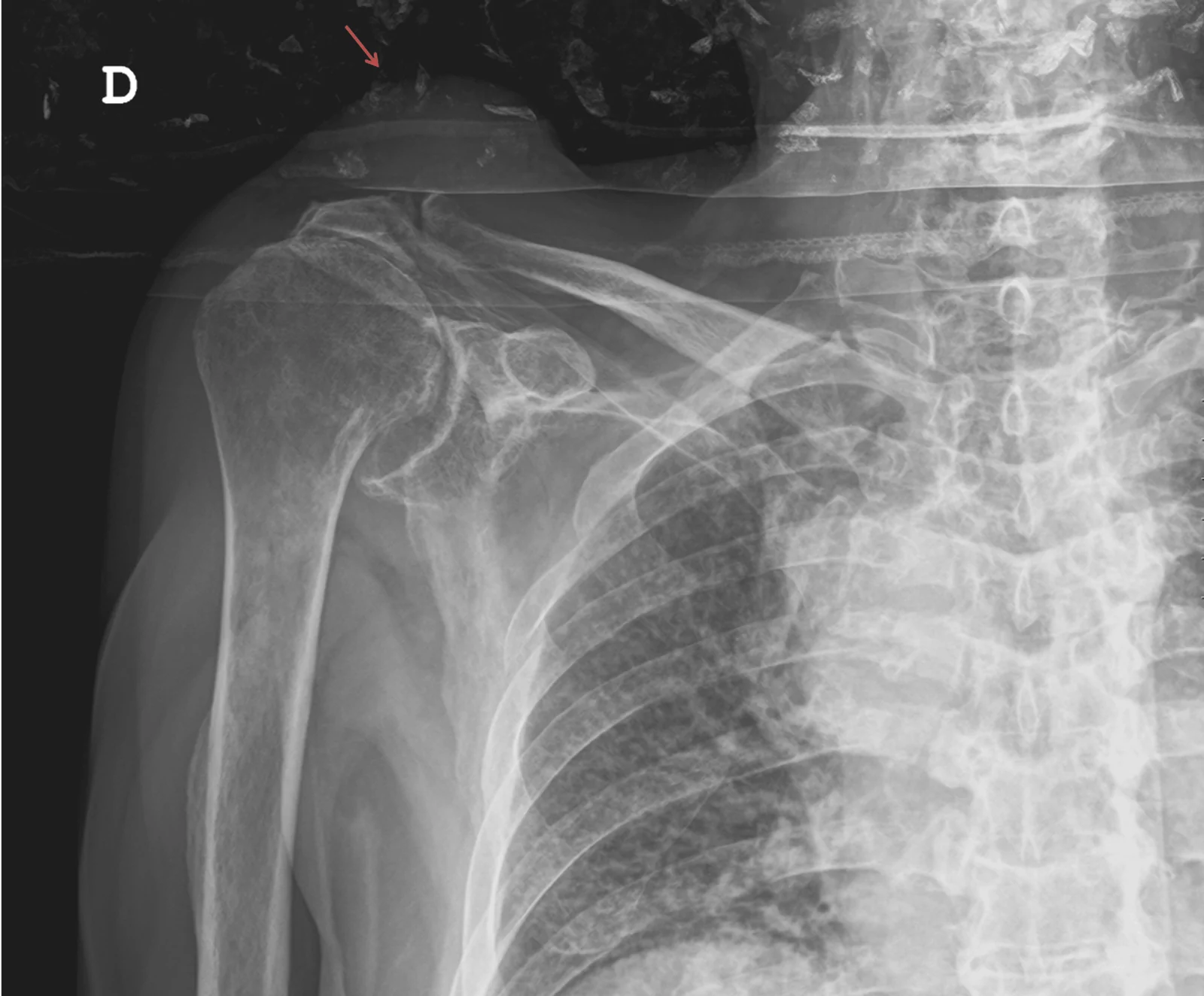
Right shoulder radiograph showing soft-tissue swelling superior to the ACJ (arrow), advanced degenerative changes of the GHJ and ACJ, superior migration of the humeral head, and narrowing of the subacromial space. ACJ, acromioclavicular joint; GHJ, glenohumeral joint

**Figure 3:**
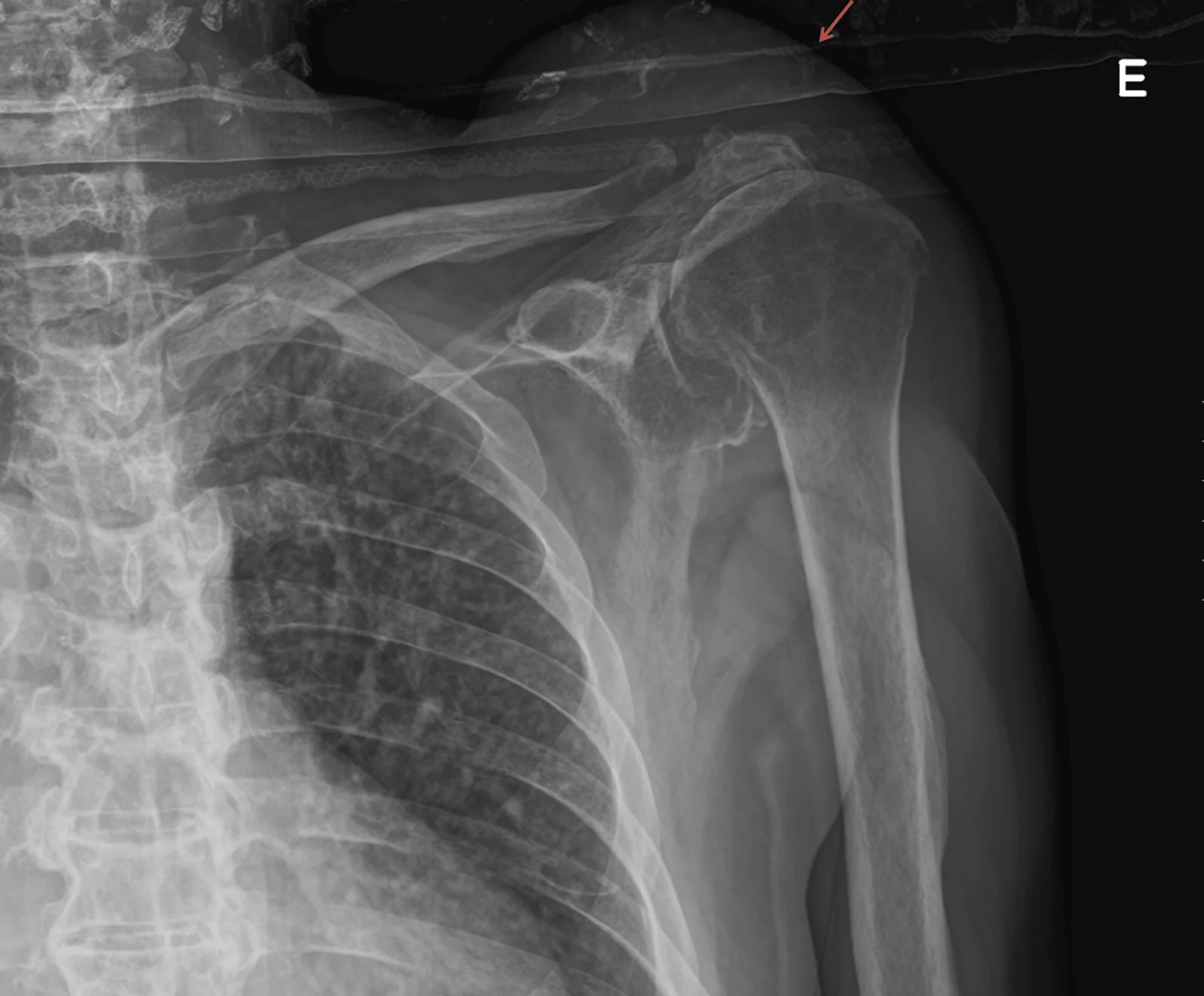
Left shoulder radiograph showing soft-tissue swelling superior to the ACJ (arrow), advanced degenerative changes of the GHJ and ACJ, superior migration of the humeral head, and narrowing of the subacromial space. ACJ, acromioclavicular joint; GHJ, glenohumeral joint

**Figure 4:**
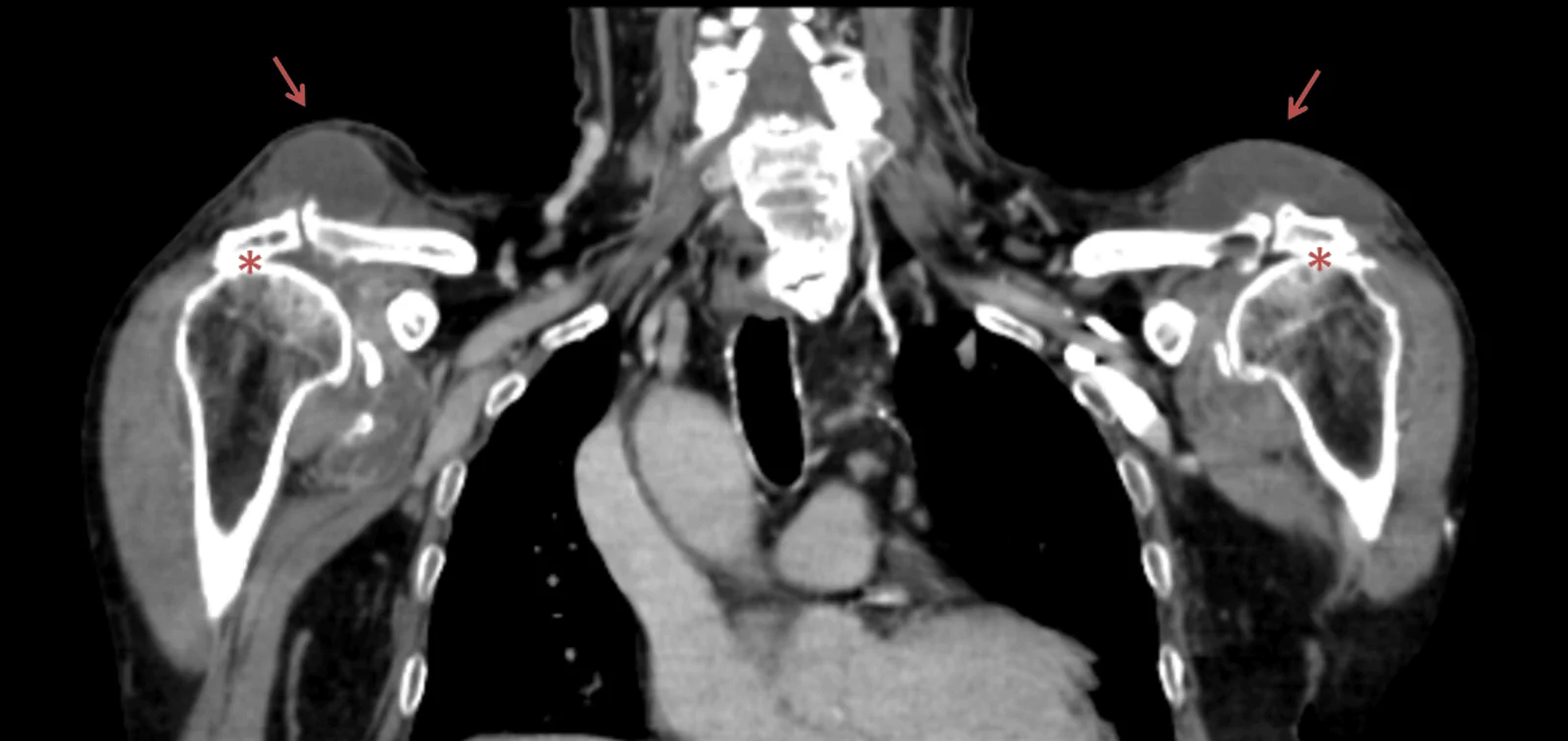
Coronal CT image of the shoulders demonstrating bilateral ACJ cysts (arrows) and imaging features suggestive of chronic rotator cuff tears (asterisks), with associated degenerative shoulder arthropathy, consistent with the GS. ACJ, acromioclavicular joint; GS, Geyser Sign

**Figure 5:**
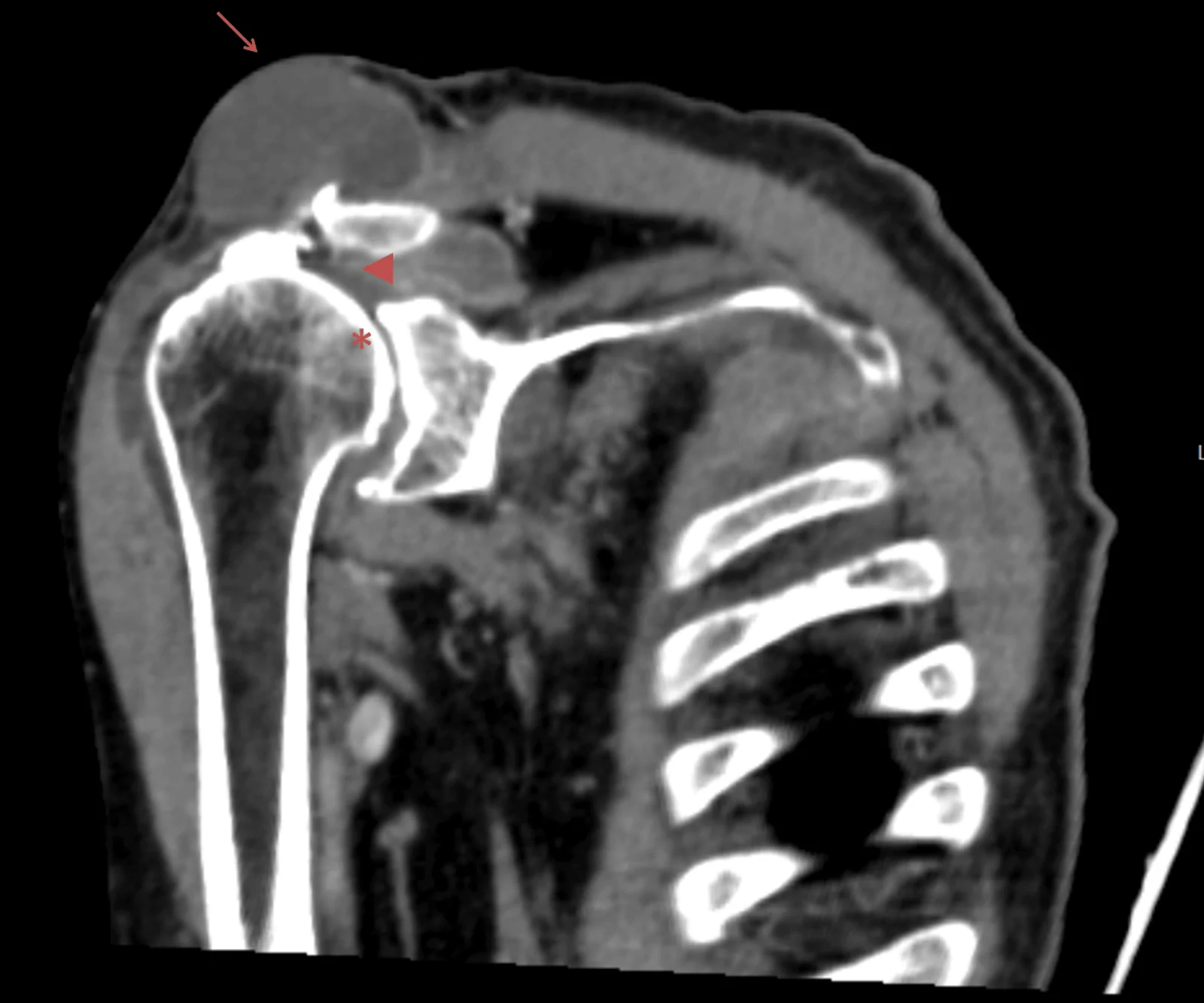
Coronal CT image of the right shoulder demonstrating an ACJ cyst (arrow), GHJ degenerative changes (asterisk), and a suspected superior fluid tract (arrowhead), compatible with GS. ACJ, acromioclavicular joint; GS, Geyser Sign; GHJ, glenohumeral joint

**Figure 6:**
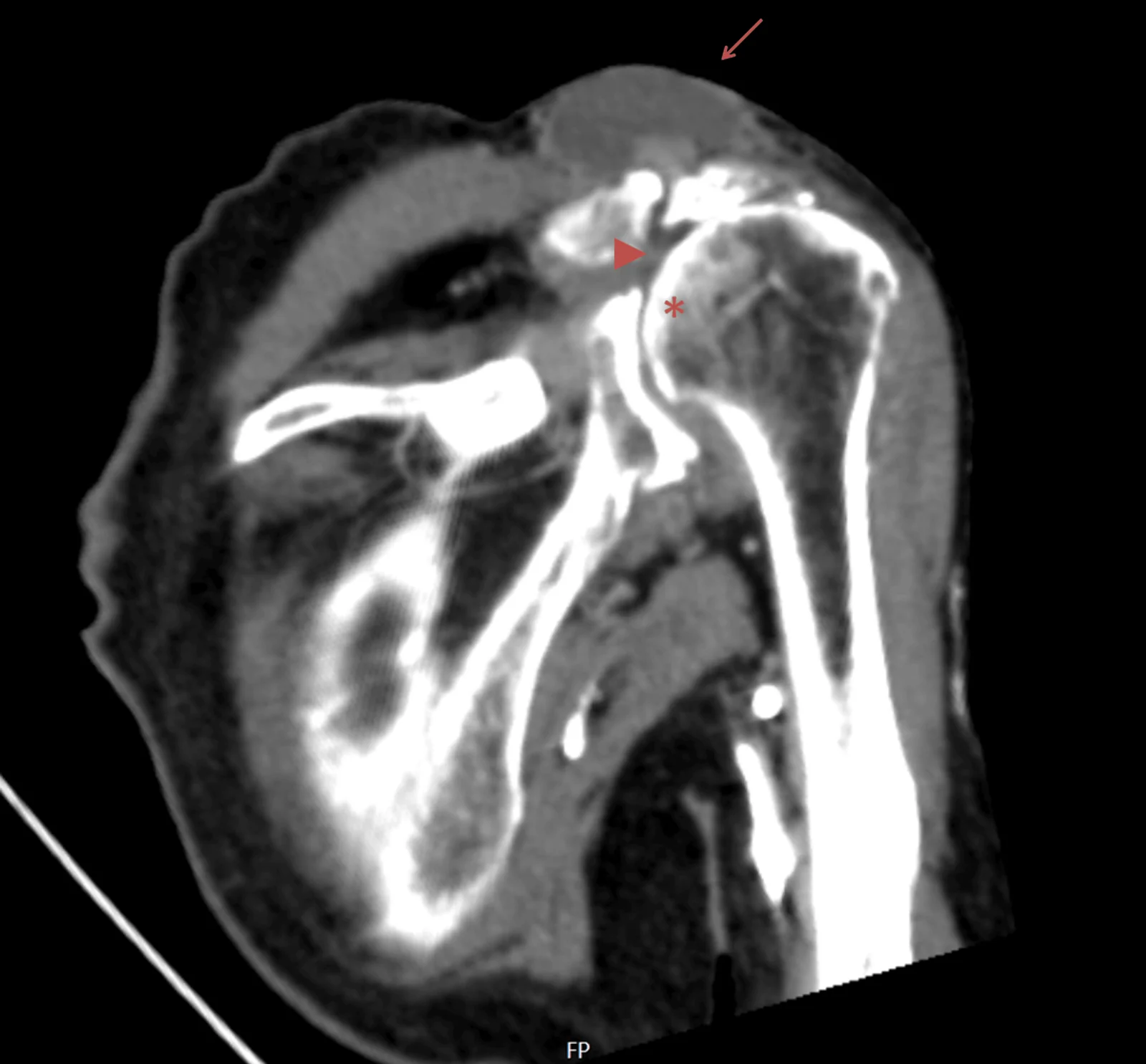
Coronal CT image of the left shoulder demonstrating an ACJ cyst (arrow), GHJ degenerative changes (asterisk), and a suspected superior fluid tract (arrowhead), compatible with the GS. ACJ, acromioclavicular joint; GS, Geyser Sign; GHJ, glenohumeral joint

A 72-year-old man presented with a two-month history of bilateral shoulder pain. The patient denied previous trauma or constitutional symptoms, including fever. His medical history was significant for GHJ osteoarthritis and rotator cuff pathology. Physical examination revealed firm and compressible swelling with marked inflammatory signs over both ACJs (Figure 1), painful and limited active and passive bilateral shoulder range of motion - particularly abduction and external rotation - and coarse glenohumeral crepitus. Shoulder radiographs (Figures 2-3) demonstrated soft tissue swelling superior to the ACJ, with marked degenerative changes involving both the GHJ and ACJ, superior migration of the humeral head with narrowing of the subacromial space - findings consistent with chronic rotator cuff arthropathy. A computed tomography (CT) scan (Figures 4, 5, 6) revealed severe omarthrosis, a complete rotator cuff tear, a large right GHJ effusion and minimal effusion on the left, significant distension of the right subacromial bursa, and a cystic image in the superior aspect of the ACJ measuring 5.4 cm on the right and 6.3 cm on the left. These findings suggested capsuloligamentous disruption and were consistent with bilateral GS. To provide symptomatic relief, ultrasound-guided cyst aspiration was performed on the right shoulder, the more symptomatic side at presentation, yielding yellow, viscous synovial fluid with 14,822 white blood cells/mm³ (89% polymorphonuclear cells), presence of calcium pyrophosphate crystals, and negative bacterial culture - findings consistent with CPPD. The patient was treated with a single injection of triamcinolone (40 mg) on the right side, with symptomatic relief sustained over six months of follow-up.

## Discussion

To the best of our knowledge, bilateral presentation of the GS is an uncommon finding in the literature, as most reported cases are unilateral and typically associated with degenerative shoulder disease. Although CPPD frequently affects the shoulder [5], ACJ cysts associated with calcium pyrophosphate crystal deposition are exceptionally rare. Cooper et al. [6] reported two cases of ACJ cysts with calcium pyrophosphate crystal deposition, both presenting with clinically evident bilateral swelling, although crystal confirmation by aspiration was obtained only on the symptomatic side in each case. Tshering Vogel et al. [7] described a series of nine unilateral ACJ cysts, including two cases with calcium pyrophosphate crystal deposition, occurring in older patients with degenerative GHJ changes and rotator cuff tears. In this context, our findings are consistent with previous reports regarding the association between GS, rotator cuff tears, and degenerative joint disease. Our case stands out for its unusual bilateral involvement, supported by symmetric clinical and imaging findings on both shoulders, and for the identification of calcium pyrophosphate crystals within the ACJ cysts, although confirmed only on the right side. These observations raise the possibility that crystal deposition may contribute to synovial inflammation and structural joint and periarticular damage [5], potentially facilitating communication between the GHJ and ACJ; however, causality cannot be established from a single case, and further studies are needed to clarify the role of crystal deposition in this process. Management strategies for ACJ cysts include both non-surgical and surgical approaches, depending on the patient’s symptoms, functional status, extension of rotator cuff tears, and comorbidities [3,4]. This case represents a type 2 ACJ cyst, in which management is particularly challenging due to its communication with the GHJ and the presence of a chronic rotator cuff tear [4]. In this patient, despite the complete rotator cuff tear, shoulder function remained relatively preserved, and a conservative approach with ultrasound-guided aspiration combined with corticosteroid injection was therefore favored. This resulted in sustained symptomatic improvement with no recurrence during follow-up, and surgical intervention was not considered at this stage. Recognition of the underlying pathophysiological context, including possible associated crystal arthropathy, is important for guiding appropriate management. This report has inherent limitations, including those of a single-case observation, which preclude broader generalization. Additionally, synovial fluid analysis confirming CPPD was performed only on the right side, and bilateral crystal deposition was therefore inferred from the symmetric clinical and imaging findings.

## Conclusions

This case illustrates a rare manifestation of a rotator cuff tear associated with GHJ osteoarthritis in a patient with CPPD. The bilateral presentation of the GS further emphasizes the unusual nature of this finding, as most reported cases are unilateral and related to advanced degenerative shoulder disease. This report highlights the potential association between crystal arthropathy and the GS and underscores the diagnostic value of cyst aspiration when clinically feasible. Awareness of a possible association between CPPD and the GS may aid diagnostic reasoning and support appropriate management decisions.
